# Supplementary material for: Gut dysbiosis is associated with acceleration of lupus nephritis
Source: Sci Rep. 2022 Jan 7;12:152. doi: 10.1038/s41598-021-03886-5 (PMC8742035; doi:10.1038/s41598-021-03886-5)
Supplement: Supplementary file 1 — Supplementary Information. [file 41598_2021_3886_MOESM1_ESM.docx]

**TITLE**: Gut Dysbiosis is Associated with Acceleration of Lupus Nephritis

**AUTHORS**:

Giancarlo R. Valiente^1^, Armin Munir^2^, Marcia L. Hart^3^, Perry Blough^2^, Takuma T. Wada^4^, Emma E. Dalan^2^, William L. Willis^2^, Lai-Chu Wu^5^, Aharon G. Freud^6^, Wael N. Jarjour2,*

**AFFILIATIONS**:

^1^Medical Scientist Training Program, The Ohio State University, Columbus, OH, USA

^2^Department of Rheumatology and Immunology, The Ohio State University Wexner Medical Center, Columbus, OH, USA

^3^IDEXX Bioanalytics, Columbia, MO, USA

^4^Saitama Medical University, Moroyama, Saitama, Japan

5Department of Biological Chemistry and Pharmacology, The Ohio State University, Columbus, OH, USA

6The James Cancer Hospital and Solove Research Institute, The Ohio State University, Columbus, OH, USA

***CORRESPONDING AUTHOR:**

Wael N. Jarjour

Department of Rheumatology and Immunology

The Ohio State University Wexner Medical Center

Work Phone: 614-366-7016

Fax: 614-366-0980

Email: wael.jarjour@osumc.edu

**Supplemental Figure 1**. SFB testing in NZM2410 mice inoculated with B6 -SFB or B6 +SFB fecal homogenates. SFB-specific 16S rDNA primers by PCR is shown at 108 base pairs. NZM2410 mice were tested at 30 weeks of age after being oral gavaged with B6 –SFB **A**. or B6 +SFB **B**. fecal homogenates at 10 weeks of age. B6 +/- SFB mice were purchased from Taconic Farms; mice were tested by the vendor and upon arrival to our vivarium for SFB.


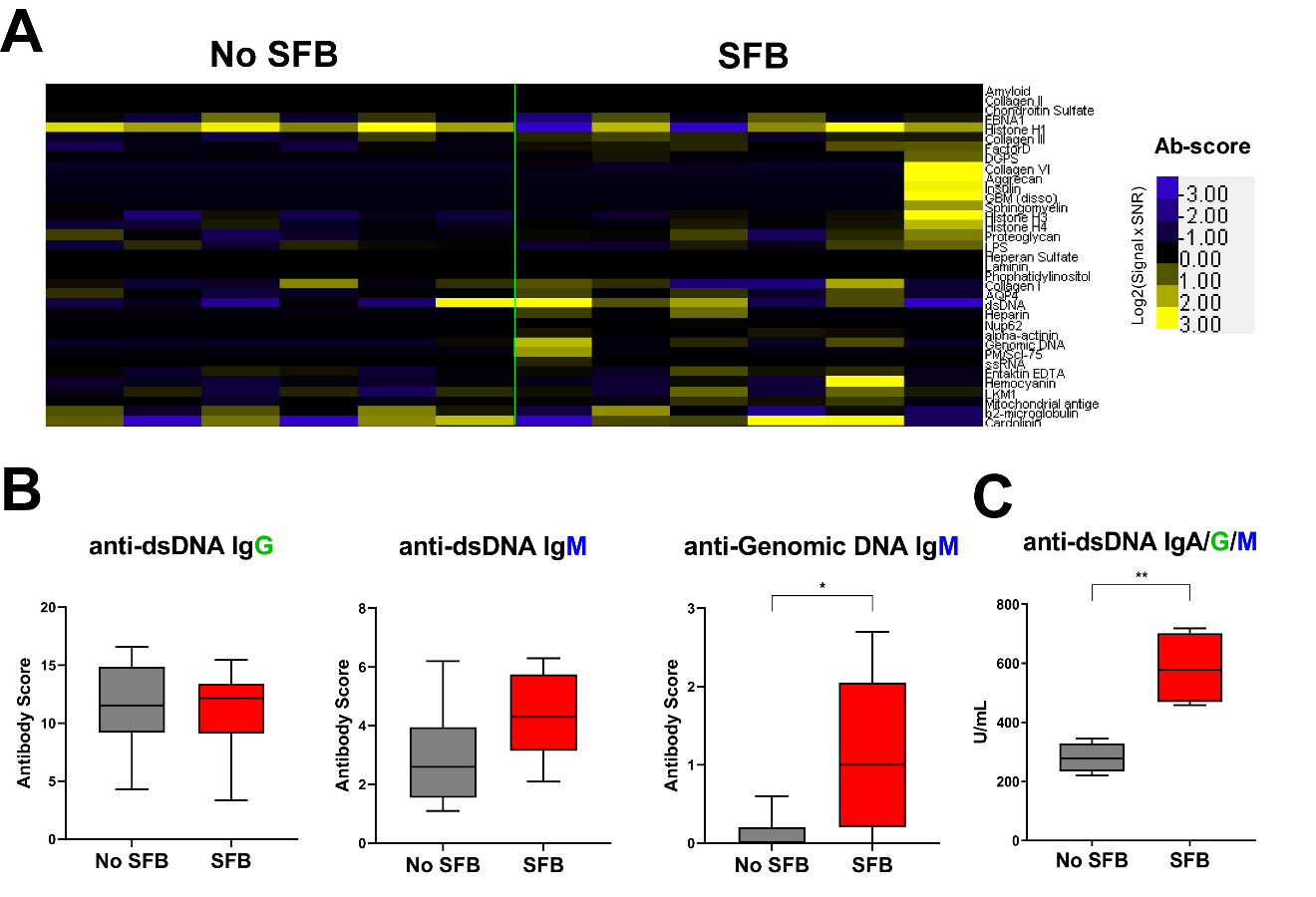


**Supplemental Figure 2.** SFB positivity is associated with antibody specificity and isotype differences in NZM2410 mice. **A**. Serum from 30 week old SFB+ and SFB− mice was applied to IgM antigen microarray; heat map legend on right. **B**. Autoantigen microarray comparison of IgG and IgM autoantibodies. **C**. Anti-dsDNA autoantibody ELISA that detects IgG, IgA and IgM isotypes. n = 6 individual mice per SFB+ and SFB− group. *p<0.05; **p<0.005.

**
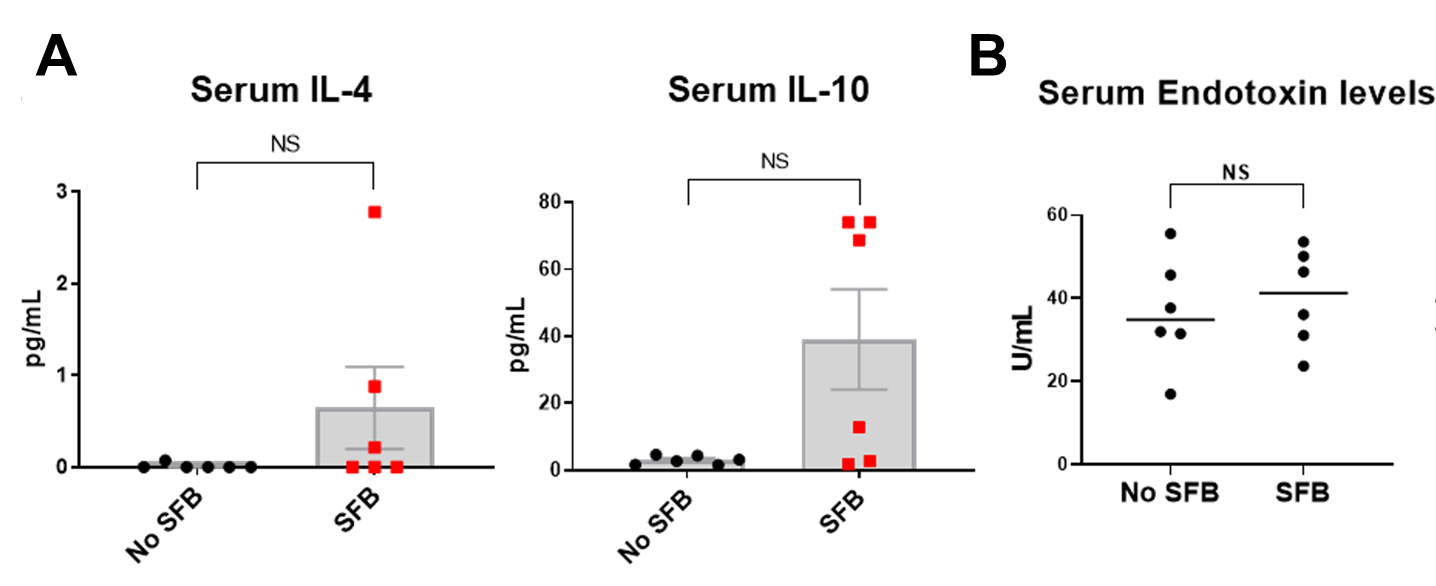
**

**Supplemental Figure 3.** M2 macrophage markers and serum endotoxin (LPS) levels are not significantly elevated in +SFB mice. **A**. IL-4 and IL-10 serum cytokines as measured by ELISA from +SFB and −SFB at 30 weeks of age. **B**. Serum LPS levels as measured from the same mice. NS = not significant (p<0.05).

**Supplemental Figure 4**. Multi-taxon 16S rRNA analysis of NZM2410 mice +/-SFB at 15 or 30 weeks of age. **A**. Principal Component Analysis (PCoA) of all operational taxonomic units (OTUs) in the four cohorts of mice. The bulk of the variability in the groups is noted in PCoA1 and PCoA2. **B**. Percent relative abundance of significant taxa at the family level from representative fecal samples of SFB positive mice at 30 weeks of age. Statistical significance determined using the Mann Whitney test (p≤0.05 statistically significant). Asterisks denote statistical significance between groups. **C**. Bar charts of relative abundance of taxa at the Firmicutes and Bacteroidetes Phylum level at 15 and 30 weeks, with and without SFB. **D**. Firmicutes to Bacteroidetes ratio between the same cohorts in A. Statistical significance was not achieved as determined by two-way ANOVA or Kruskal-Wallis, depending on normality of data as determined by Shapiro-Wilk normality testing and Benjamini-Hochberg correction for multiple testing (p≤0.05 statistically significant).


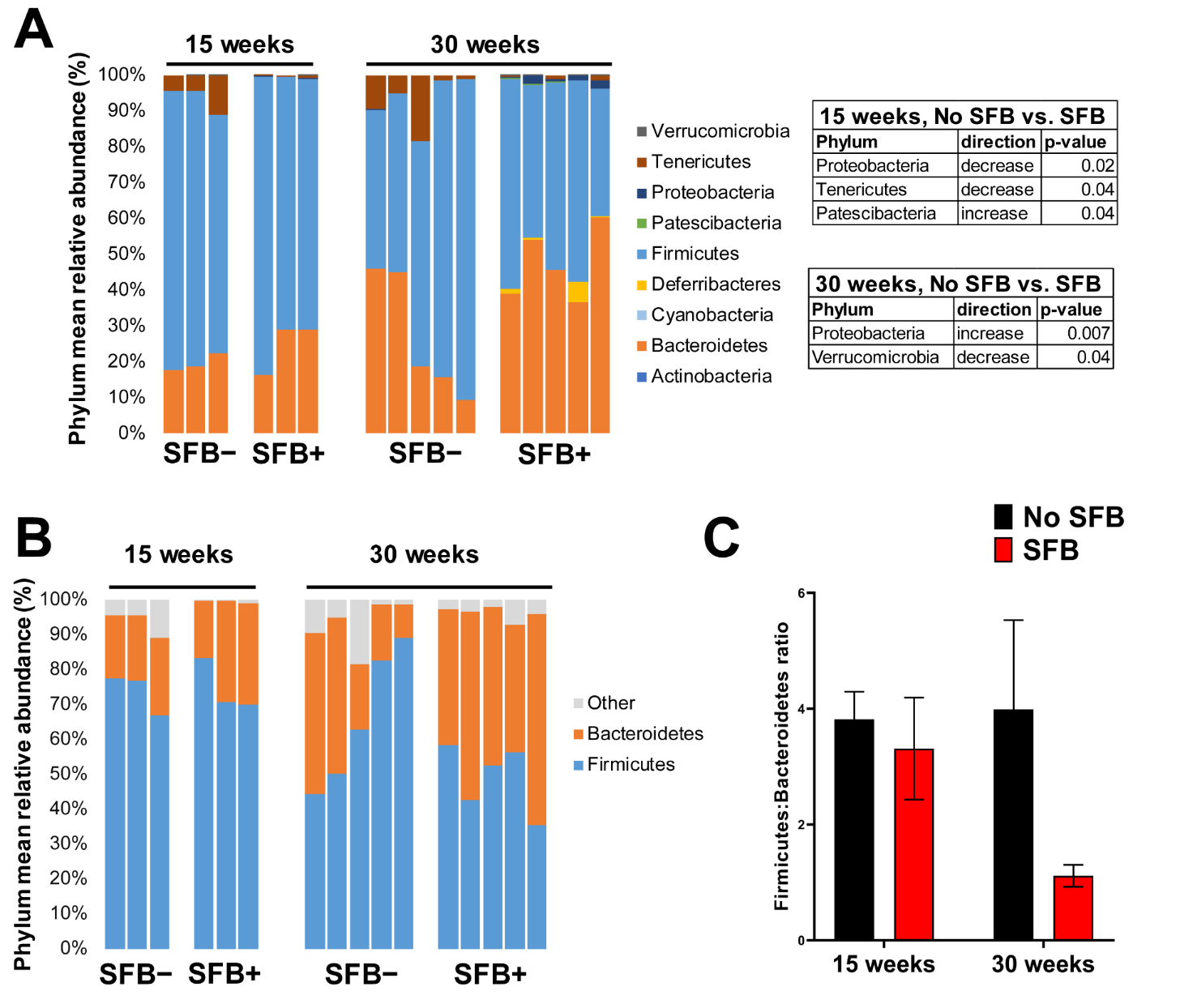


**Supplemental Figure 5**. Phyla 16S rRNA analysis of NZM2410 mice +/−SFB at 15 or 30 weeks of age. Bar charts of relative abundance of taxa at the phyla level at 15 and 30 weeks, with and without SFB with accompanying statistical information.

**Supplemental Figure 6**. *Ruminococcus torques* group from 16S rDNA analysis of +SFB and –SFB NZM2410 mice at 30 weeks of age (n = 3-5 mice per group). **A**. Comparison of *R. torques* species in +SFB and −SFB NZM2410 mice at 30 weeks of age. **B**. Phylogenetic comparison of *R. torques* and *R. gnavus* based on PATRIC database analysis (67). *p≤0.005.
